# Three new species of *Gymnopus* and *Mycena* (Agaricales, Basidiomycota) from Northwestern China

**DOI:** 10.3389/fmicb.2024.1487598

**Published:** 2024-11-29

**Authors:** LongFei Fan, BiYue Wang, TianFu Ma, Bin Li, JianWei Ma, XuTao Lei, NingHui Bao

**Affiliations:** College of Plant Protection, Gansu Agricultural University, Lanzhou, China

**Keywords:** *Gymnopus* sect. Impudicae, *Mycena*, macrofungi, morphology, phylogenetic analyses

## Abstract

Members of the genera *Gymnopus* and *Mycena* are vital for litter decomposition in tropical and humid temperate forests. In this study, the difference in morphological features among *Gymnopus gansuensis*, *Gymnopus subsepiiconicus*, and *Mycena glabera* was confirmed by DNA data. *Gymnopus gansuensis* and *Gymnopus subsepiiconicus* showed separate relationships with other species in the ITS and nLSU combined dataset utilized for the phylogeny of *Gymnopus* sect. *Impudicae*. In addition, *Gymnopus gansuensis* is characterized by pileus honey yellow at the center, margin pinkish buff to buff, stipe pinkish buff to fuscous, and basidiospores elliptic to briolette. *Gymnopus subsepiiconicus* is characterized by pileus clay buff to grayish brown at the center, margin pinkish buff to fawn, stipe dark brown to fuscous, and basidiospores elliptic. Based on the combined dataset of the ITS and TEF-1α, *Mycena glabera* has been detected as a separate lineage in the phylogenetic studies of *Mycena* sect. *Calodontes*. The ecological behaviors of the new species are described with illustrations.

## 1 Introduction

*Gymnopus* (Pers.) Roussel was classified as a member of the family Omphalotaceae by Antonín and Noordeloos (2010). There are 448 records of this genus in the Index Fungorum^1^ and 483 records in the MycoBank database.^2^ Approximately, 300 species of *Gymnopus* have been validly published (Coimbra et al., 2015; Kirk et al., 2008; Deng et al., 2016).

Antonín and Noordeloos (1997) proposed that *Gymnopus* should be classified based on white to cream-colored spore print, a non-insititious stipe, and different types of pileipellis. These authors divided the genus into three taxonomic groups (sect. *Gymnopus*, sect. *Vestipedes*, and sect. *Levipedes*) and four subgroups (subsect. *Impudicae*, subsect. *Vestipedes*, subsect. *Levipedes*, and subsect. *Alkalivirentes*). Further taxonomic revisions by Mata et al. (2006) proposed to rename *Marasmius* sect. *Peronati* to *Gymnopus* subsect., and Ovrebo’s (Mata and Ovrebo, 2009) establishment of *Gymnopus* sect. *Androsacei*.

Among the studies with molecular analysis, molecular research has been used to investigate the phylogenetic relationship within *Gymnopus*. Using nLSU sequences, Moncalvo et al. (2002) confirmed that *Gymnopus*, *Lentinula*, *Rhodocollybia,* and some other fungi belong to the Omphalotaceae clade. Mata et al. (2004) demonstrated a close relationship between *Gymnopus* and *Marasmiellus* Murrill, noting that *Marasmiellus juniperinus* Murrill, the type species of *Marasmiellus*, was included in *Gymnopus* sect. Levipedes. Wilson and Desjardin (2005) supported these findings, affirming that *Gymnopus* belongs to Omphalotaceae. Antonín and Noordeloos raised species of *Gymnopus* with a distinctly unpleasant smell to the sectional rank of *Gymnopus* sect. *Impudicae*. According to modern definition, species in *Gymnopus* sect. *Impudicae* are characterized by the pileipellis composed of cylindrical hyphae and a distinctive strong, unpleasant, onion, sewage, or rotten cabbage-like smell (Ryoo et al., 2016). Hu et al. (2024) conducted a comprehensive study about *Gymnopus* s. l. based on 527 gymnopoid collections from China. sect. *Levipedes* and sect. *Impudicae* were recognized as independent genera in Omphalotaceae, and six sections were accepted in *Gymnopus* depending on Melinda’s reagent. However, to be conservative, we still use the concept of sect. *Impudicae* due to this new classification framework, which needs further validation, and some species complexes still exist.

The genera *Gymnopus* and *Mycena* (Pers.) Roussel have also been studied in China. Teng (1963) reported the first (under Collybia) *Gymnopus* species in China as *Collybia radicans* P. Kumm. [= *Hymenopellis radicata* (Relhan) R.H. Petersen]. Overall, 19 taxa of *Gymnopus* were found in Southern China by Deng (2016). Ongoing research on *Gymnopus* has led to the identification of 57 species of *Gymnopus* s. l. based on morphological characteristics and molecular datasets and 32 species of *Gymnopus* s. str. described from China (Deng et al., 2016; Dai et al., 2010; Li et al., 2021a,b; Mešic et al., 2011; Wang et al., 2020; Sun et al., 2021; Hu et al., 2022; Hu et al., 2024). *Mycena*, one of the largest genera in the order Agaricales, comprises nearly 600 species distributed worldwide (He et al., 2020; Wei et al., 2024; Zhang et al., 2024). It has been separated into seven clades based on ITS+nLSU+SSU and 23 sections according to morphological feathers (Na et al., 2022; Cortés-Pérez et al., 2023). *Mycena* sect. *Calodontes* (Fr. ex Berk.) Quél. is characterized by its hygrophanous pileus exhibiting pinkish, reddish, purplish to brownish, intervenose lamellae, smooth cheilocystidia and pleurocystidia (if present), and frequently amyloid basidiospores (Cortés-Pérez et al., 2023). Pileipellis types and cheilocystidia characteristics may be vital for delimiting brownish *Mycena* species (Wei et al., 2024). As a significant group within the order Agaricales, *Mycena* encompasses at least 500 species globally, with most occurring in the northern temperate zone, primarily as saprophytes (Liu et al., 2022). These small mushrooms hold edible and medicinal value, or they may contain trace toxins and serve as germination fungi in the cultivation of Bl. With the application of multigene phylogeny in taxonomic studies, an increasing number of mushroom species are being reported from various regions around the world (Cortés-Pérez et al., 2023; Liu et al., 2022).

During our investigations of macrofungi in northwestern China, some specimens of *Gymnopus* and *Mycena* were collected. *Gymnopus* sect. *Impudicae* and *Mycena* sect. *Calodontes* phylogenetic analyses were performed in the present study using the combined datasets of ITS + nLSU and ITS + TEF-1α, respectively. Following morphological and molecular evidence, three previously unidentified species were discovered. Detailed descriptions and illustrations of these species are provided.

## 2 Materials and methods

### 2.1 Morphological examined

The specimens examined in this study were gathered in Northwest China, and their macroscopic features were recorded in fresh as the basis for the macromorphological descriptions, including host, ecological behaviors, geographical coordinates, location, altitude, collector, and date. Photographs of fresh basidiocarps and habitat information were taken in the field. All samples studied in this study were dehydrated and stored in the herbaria at Gansu Agricultural University (MHGAU, China). Micromorphological characters were observed from dried materials and examined with a light microscope (Nikon Eclipse E 80i microscope, Nikon, Tokyo, Japan) following the general techniques used in past research (Hu et al., 2022). In the descriptions, the abbreviations mean: IKI = Melzer’s reagent, IKI– = negative reaction in Melzer’s reagent, KOH = 5% potassium hydroxide, CB = Cotton Blue, CB+ = cyanophilous, CB− = acyanophilous, L = mean spore length, W = mean spore width, Q = variation in the L/W ratio between the specimens studied, and *n* = number of spores measured from the number of samples. The microscopic features were examined and described in 1% Congo red aqueous solution when necessary (Wei et al., 2024).

### 2.2 DNA extraction, PCR amplification, and sequencing

A CTAB plant genome rapid extraction kit-DN14 (Aidlab Biotechnologies Co., Ltd.) was utilized to extract DNA from dried specimens. With slight modifications, the manufacturer’s instructions for the PCR were carried out when employing the extracted DNA (Hu et al., 2022). The ITS gene region was amplified using the primer pairs ITS5/ITS4 and LR0R/LR7 for the nLSU gene region and 983F/1567R for the TEF-1α gene region (Gardes and Bruns, 1993; Cubeta et al., 1991). The PCR procedure for ITS and TEF-1α was as follows: initial denaturation at 95°C for 3 min, followed by 35 cycles at 94°C for 40 s, 56°C (ITS) or 55°C (TEF-1α) for 45 s and 72°C for 1 min, and a final extension of 72°C for 10 min (Li et al., 2022). The PCR procedure for nrLSU was as follows: initial denaturation at 94°C for 1 min, followed by 35 cycles at 94°C for 30 s, 50°C for 1 min, 72°C for 1.5 min, and a final extension of 72°C for 10 min (Li et al., 2022). The PCR products were purified and sequenced at the Xian Genomics Institute in China using the same pair of primers. Every newly produced sequence was uploaded to GenBank (Tables 1, 2).

**Table 1:** A list of species, specimens, and GenBank accession numbers of sequences used in this study.

Species	Specimen no.	Locality	GenBank accession no.	
			ITS	nrLSU
*Gymnopus alliifoetidissimus*	GDGM76695T	China	NR174903	NG079661
				NG_079661
*G. alliifoetidissimus*	HMJAU61026	China	OQ597033	OQ594443
*G. atlanticus*	URM87728	Brazil	KT222654	KY302698
				KY302698
				KY302698
*G. atlanticus*	R1OTU520	Brazil	ON562324	-
*G. aurantiipes*	AWW118	United States	AY263432	AY639410
*G. aurantiofuscus*	HGASMF017024T	China	PP151504	PP151551
*G. aurantiofuscus*	HGASMF017009	China	PP151518	PP151562
*G. barbipes*	TENN67855	United States	NR152901	-
			NR_152901	
*G. bicolor*	AWW116	United States	AY263423	AY639411
*G. brunneostipitatus*	HMJAU 60412 T holotype	China	PP151535	PP639544
*G. brunneostipitatus*	HMJAU 60448	China	PP657584	PP639545
*G. chowii*	HMJAU 60415 T	China	PP151495	PP151548
*G. chowii*	HMJAU 60416	China	PP151496	-
*G. conifericola*	HMJAU 60413 T	China	PP151542	PP151571
*G. conifericola*	HMJAU 60413	China	PP151543	-
*G. densilamellatus*	BRNM714927	Korea	KP336685	KP336694
*G. densilamellatus*	HMJAU61015	China	ON259034	ON259045
*G. dysodes*	TENNF61125	United States	KY026666	-
*G. foetidus*	TENN59259	Austria	KJ416259	-
*G. foetidus*	TENN61221	United States	KJ416258	-
*G. fuscus*	GDGM70487	China	PP151516	PP151560
*G. fuscus*	GDGM70488	China	PP151517	PP151561
*G. iocephalus*	TFB6520	United States	DQ449984	KY019630
*G. iocephalus*	DukeRV94154	United States	DQ449986	-
*G. iodes*	HGASMF0110068T	China	OM970869	-
		China		
*G. iodes*	HGASMF0110069	China	OM970868	-
*G. impudicus*	JVG11305312	Spain	LT594120	LT594120
*G. impudicus*	BRNM714849	Czech Republic	LT594119	LT594119
*G. montagnei*	JMCR143	United States	DQ449988	AF261327
*G. montagnei*	URM87715	Brazil	KT222652	KX958400
*G. polyphyllus*	TENN62814H1	United States	FJ596894	-
*G. polyphyllus*	TENN62814H2	United States	FJ596895	-
*G. sepiiconicus*	AWW126	United States	AY263449	AY639427
*G. sinopolyphyllus*	HMJAU60386T	China	OM970872	OM970872
*G. sinopolyphyllus*	HMJAU60387	China	OM970871	OM970871
			OM970871	
*G. similis*	HMJAU61053	China	OQ597050	OQ594460
*G. similis*	BRNM714981	Korea	KP336690	KP336697
				KP336697
*G. salakensis*	AWW29	United States	AY263447	-
*G. variicolor*	BRNM714959	Korea	LT594121	KP348011
*G. variicolor*	BRNM781307T	Korea	KX926134	-
*G. vitellinipes*	AWW127	United States	AY263429	AY639432
***G. gansunensis***	**FLF541**	**China**	**PP911508**	**PP907040**
***G. subsepiiconicus***	**FLF393**	**China**	**PP911505**	**PP907039**
***G. subsepiiconicus***	**FLF739**	**China**	**PP911506**	**-**
***G. subsepiiconicus***	**FLF757**	**China**	**PP911507**	**-**
*Mycetinis alliaceus*	TENNF55630	Russia	KY696752	KY696752
*Mycetinis scorodonius*	TENNF53474	United States	KY696748	KY696748

New sequences are shown in bold.

**Table 2:** A list of species, specimens, and GenBank accession numbers of sequences used in this study.

Species	Specimen no.	Locality	GenBank accession no.	
			ITS	Tef-1
*Mycena aff. pura*	TL8052	Ecuador	FN394623	KF723641
				NG_079661
*M. aff. pura*	TL9433	Ecuador	FN394622	KF723642
	TL9450			
	TL9678			
	BAP594			
*M. aff. pura*	TL9450	Ecuador	KJ144653	KF723643
	TL9450			KY302698
	TL9678			KY302698
	BAP594			
*M. aff. pura*	TL9678	Ecuador	FN394621	KF723644
*M. brunneoviolacea*	BAP594	Africa	MH414546	-
*M. cahaya*	ACL134	Malaysia	KF537248	-
*M. calongei*	AH56035	Spain	OQ633199	-
*M. calongei*	AH56036T	Spain	OQ633200	-
*M. cf. pura* I	CBH039	Denmark	FN394588	KF723634
*M. cf. pura* II	CBH105	Denmark	FN394581	KF723625
*M. cf. pura* II	CBH366	Denmark	FN394572	KF723627
*M. cf. pura* III	CBH019	Denmark	FN394605	KF723629
*M. cf. pura* III	CBH022	Denmark	FN394574	KF723630
*M. cf. pura* IV	CBH410	Denmark	FN394595	KF723621
*M. cf. pura* IV	JV06979	Denmark	FN394585	KF723622
*M. cf. pura* V	CBH226	Denmark	FN394604	KF723618
*M. cf. pura* V	TL5614	Denmark	FN394602	KF723620
*M. cf. pura* VI	BAP132	USA	FN394561	KF723614
*M. cf. pura* VII	IS10/11/200	USA	FN394611	–
*M. cf. pura* VIII	CBH216	Denmark	FN394598	KF723616
*M. cf. pura* VIII	CBH402	Denmark	FN394599	KF723617
*M. cf. pura* IX	CBH166	Denmark	FN394607	KF723655
*M. cf. pura* IX	CBH358	Denmark	FN394608	KF723656
*M. cf. pura* X	BAP165A	USA	FN394563	KF723652
*M. cf. pura* XI	CBH187	Sweden	FN394564	KF723632
*M. cf. pura* XI	CBH386	Denmark		KF723633
*M. diosma*	CBH400	Denmark	FN394617	KF723653
*M. diosma*	LK1191/2000	Germany	FN394619	KF723654
*M. dura*	10,315	Austria	FN394560	KF723648
***M. glabra***	**FLF449**	**China**	**PP949212**	**PP967101**
***M. glabra***	**WBY449**	**China**	**PP949213**	**PP967102**
*M. lammiensis*	TUR165927	Finland	FN394552	KF723651
*M. luceata*	ACP2116	Mexico	OR233614	OR233755
*M. luceata*	ACP2126	Mexico	OR233613	OR233754
*M. luciferina*	ACP2114T	Mexico	OR233612	–
*M. lucisnieblae*	ACP2140	Mexico	OR233610	OR233752
		China		
*M. lucisnieblae*	ACP2139	Mexico	OR233611	OR233753
*M. luxmanantlan*	ACP2160	Mexico	OR233603	OR233747
*M. luxmanantlan*	ACP2159	Mexico	OR233604	OR233748
*M. pearsoniana*	CBH068	Germany	FN394614	KF723645
		Germany		
*M. pearsoniana*	LK880/2002	Germany	FN394613	KF723647
*M. pelianthina*	CBH015	Denmark	FN394549	KF723649
*M. pelianthina*	CBH016	Denmark	FN394547	KF723650
*M. polycystidiata*	FFAAS0417T	China	ON427731	ON468469
*M. polycystidiata*	FFAAS0418	China	ON427732	ON468470
*M. rosea*	CBH097	Denmark	FN394556	KF723635
*M. rosea*	CBH409	Denmark	FN394551	KF723637
*M. rufobrunnea*	FFAAS0415	China	ON427729	ON468467
*M. rufobrunnea*	FFAAS0416T	China	ON427730	ON468468
*M. seminau*	ACL136	Malaysia	KF537250	-
			OM970871	
*M. seminau*	ACL308	Malaysia	KF537252	-
*M. shengshanensis*	FFAAS0424T	China	ON427739	ON468477
				KP336697
*M. shengshanensis*	FFAAS0425	China	ON427740	ON468478
*M. sinar*	ACL092	Malaysia	KF537247	-
*M. sinar*	ACL135T	Malaysia	KF537249	-
*M. sinar* var. *tangkaisinar*	ACL307T	Malaysia	KF537251	-
*M. sophiae*	ACP2157T	Mexico	OR233606	OR233749
*M. sophiae*	ACP2161	Mexico	OR233605	OR233757
*M. subulata*	FFAAS0419	China	ON427735	ON468473
*M. subulata*	FFAAS0423T	China	ON427737	ON468475
*M. yuezhuoi*	FFAAS0344	China	MW581490	MW882249
*M. yuezhuoi*	FFAAS0347	China	MW581493	MW882252
*M. meliigena*	39	Italy	JF908423	-
*M. meliigena*	39d	Italy	JF908429	-
*M. arcangeliana*	252b	Spain	JF908401	-
*M. arcangeliana*	252f	Spain	JF908402	-

New sequences are shown in bold.

### 2.3 Phylogenetic analyses

A combined ITS + nLSU dataset was used to examine the phylogenetic relationship of *Gymnopus*, and for this, *Mycetinis scorodonius* (Fr.) A.W. Wilson & Desjardin and *Mycetinis alliaceus* (Jacq.) Earle ex A.W. Wilson & Desjardin were selected as the out-groups following the study by Li et al. (2022). The combined ITS + TEF-1α dataset was used to confirm the phylogenetic relationship of *Mycena*, and following the study by Na et al. (2022), *M. meliigena* (Berk. & Cooke) Sacc. and *M. arcangeliana* Bres were selected as the out-group. MAFFT 7 was used to align the datasets (Ryoo et al., 2016), and manual adjustments were made using BioEdit (Katoh and Standley, 2013). The partition homogeneity test (PHT) (Hall, 1999) of the two-gene dataset was tested by PAUP v. 4.0b10, respectively (Farris et al., 1994) under 1,000 homogeneity replicates. Alignments were spliced using Mesquite v.3.2. The best-fit model of nucleotide evolution for the datasets was selected, respectively, with Akaike’s information criterion (AIC) using MrModeltest 2.3 (Swofford, 2002; Guindon and Gascuel, 2003; Darriba et al., 2012). Phylogenetic analyses were conducted according to the previous study (Hu et al., 2022).

Using the heuristic search, maximum parsimony (MP) analysis was carried out using PAUP*version 4.0b10. Gaps were considered as missing data, and all characters were given the same weight. This study chose the heuristic search option to determine the trees, which included TBR branch switching and 1,000 random sequence additions. All parsimonious trees were preserved, zero-length branches were collapsed, and the maximum number of trees was set to 5,000. A bootstrap analysis with 1,000 replicates was used to test the clade’s robustness (Posada and Crandall, 1998). For each maximum parsimonious tree (MPT), descriptive tree statistics including tree length (TL), consistency index (CI), retention index (RI), rescaled consistency index (RC), and homoplasy index (HI) were computed. RAxmL v.7.2.8 was used to carry out maximum likelihood (ML) analysis with a GTR + G + I model (Felsenstein, 1985). The computer calculated all model parameters, but only the best maximum likelihood tree from each search was retained. The best-fit evolution model for Bayesian inference (BI) was used utilizing MrModeltest 2.3 (Stamatakis, 2006; Posada and Crandall, 1998). MrBayes 3.2.6 was used for BI, conducting two distinct runs, each starting from random trees with four independent chains running at the same time, doing 2 million repeats, and sampling a tree every 100 generations through the online platform CIPRES Science Gateway (www.phylo.org, accessed on 23 April 2024) (Nylander, 2004). The sequence alignment was deposited at TreeBase (*Gymnopus* submission ID: 31485; *Mycena* submission ID: 31513). A majority rule consensus tree of all the remaining trees was determined, and the first 25% of the sampled trees were eliminated as burn-in. Branches were considered significantly supported when the bootstrap supports for MP and ML were greater than or equal to 50%, and the Bayesian inference (BI) was greater than or equal to 0.95. FigTree v1.4.2 was used for the visualization of phylogenetic trees.

## 3 Results

### 3.1 Phylogenetic analyses

The sequences involved in the ITS + nLSU dataset of *Gymnopus* were obtained from 47 specimens representing 28 species. The dataset contains 47 ITS and 26 nLSU sequences; among them, four ITS and two nLSU sequences are newly generated. The dataset contained 1,217 characters, including gaps (418 characters for ITS, 799 characters for nLSU), with 930 characters constant, 55 variable and parsimonious uninformative, and 232 parsimonious informative. The maximum parsimony analysis of *Gymnopus* produced 4,953 equally parsimonious trees (TL = 578, CI = 0.633, RI = 0.822, RC = 0.521, HI = 0.367). The GTR + I + G models were selected as the most effective models for every region of the combined ITS + nLSU sequence dataset, and they were used in the Bayesian analysis. The topology obtained from MP and Bayesian analysis was similar to that of ML analysis. The Bayesian analysis yielded a topology that was concordant, with an average split frequency standard deviation of 0.005966. Only the ML tree is shown in Figure 2; bootstrap support values for MP and ML ≥50% and BI ≥0.95 are noted at the nodes, respectively.

**Figure 1:**
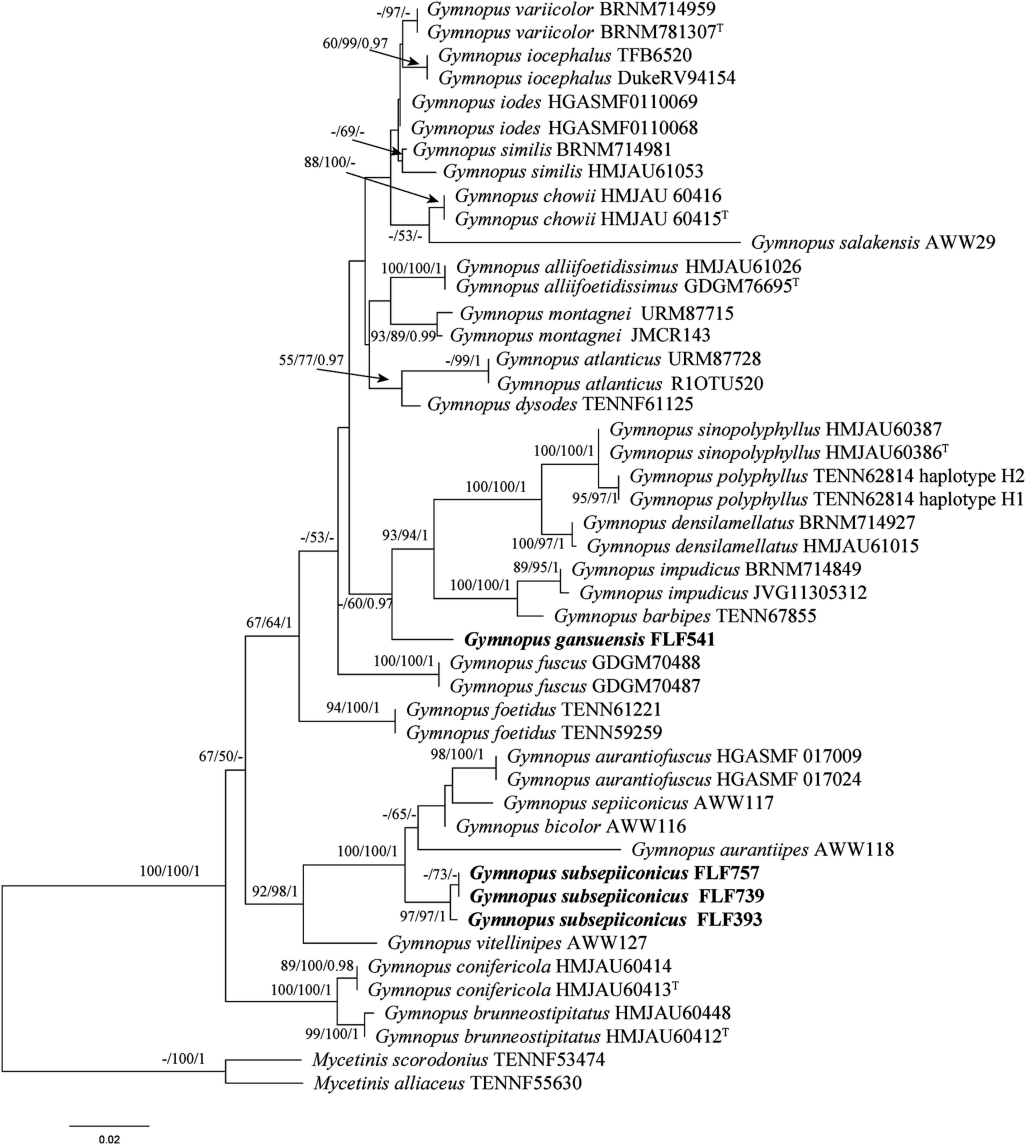
Maximum likelihood (ML) tree of species in *Gymnopus* based on the ITS + nLSU dataset. Bootstrap support values for MP and ML ≥50% and BI ≥0.95 are noted at each node, respectively. The new sequences are in bold.

The BLAST results show that the specimen FLF541 (*Gymnopus gansuensis*, holotype) is similar to *Gymnopus* sp. (taxon: 3341947), *G. barbipes* (taxon: 1460870), and *G. impudicus* (taxon: 206322), with a sequence similarity of 91.87%–95.73% from NCBI based on ITS with the top 10. *Gymnopus subsepiiconicus* (FLF393, Holotype) has a sequence similarity with *G. erythropus* (taxon: 230774), *G. longisterigmaticus* (taxon: 2935534), and *G. longus* (taxon: 2906493) ranging from 95.66% to 96.06% based on ITS with top 10.

**Figure 2:**
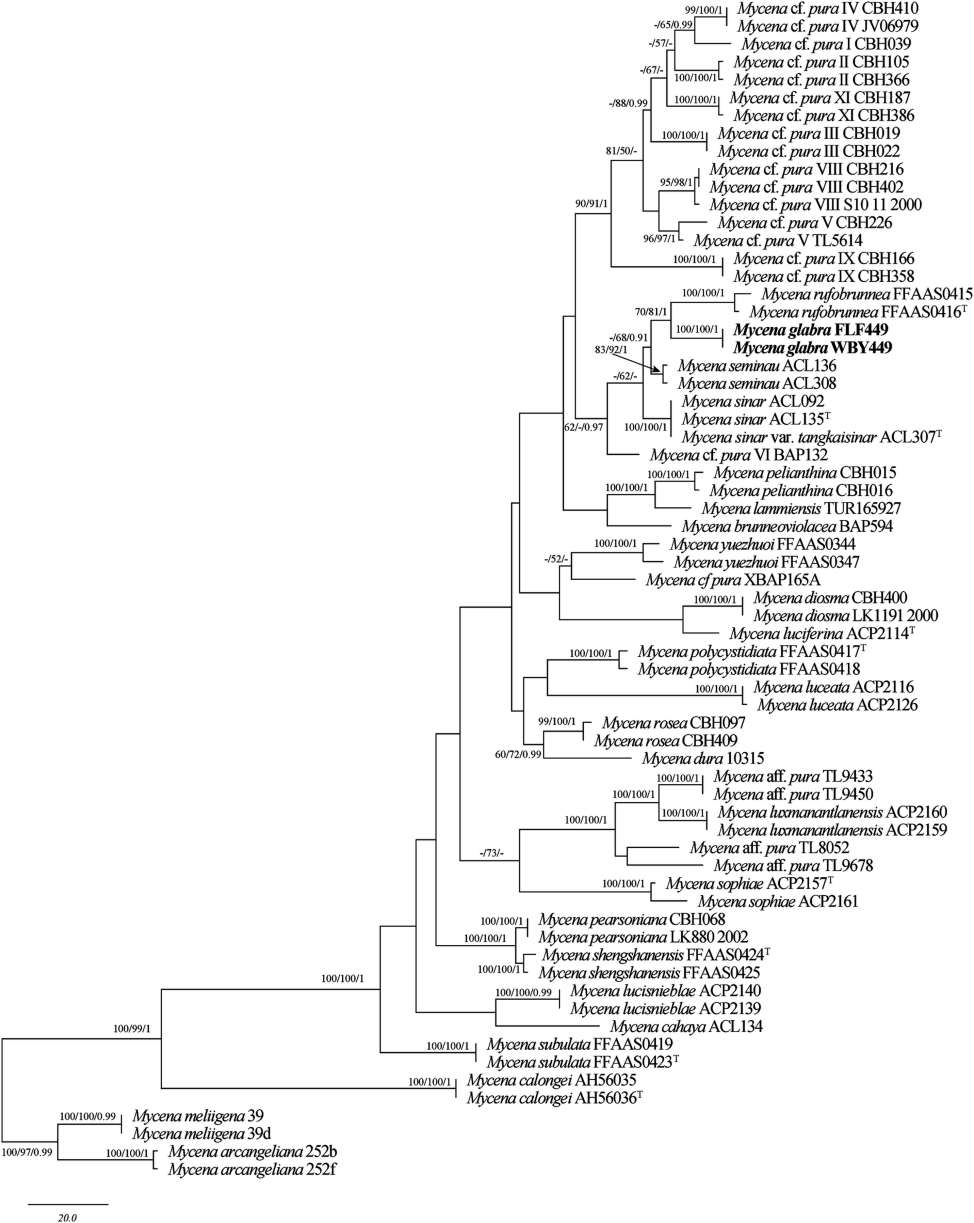
Maximum parsimony (MP) tree of species in *Mycena* based on the ITS + TEF-1α dataset. Bootstrap support values for MP and ML ≥50% and BI ≥0.95 are noted at each node, respectively. The new sequences are in bold.

The ITS + nLSU phylogenetic tree (Figure 2) confirmed the affinities of *Gymnopus gansuensis* and *Gymnopus subsepiiconicus* within *Gymnopus*, and both of them formed distinct, well-supported lineages (Figure 2).

The sequences involved in the ITS + TEF-1α dataset of *Mycena* were obtained from 66 specimens representing 37 taxa. The datasets contained 65 ITS and 51 TEF-1α sequences; among them, two ITS and two nLSU sequences are newly generated. The dataset contained 1,088 characters, including gaps (710 characters for ITS, 378 characters for TEF-1α), with 628 characters constant, 45 variable and parsimonious uninformative, and 415 parsimonious informative. The maximum parsimony analysis of *Mycena* produced 201 equally parsimonious trees (TL = 1,054, CI = 0.583, RI = 0.806, RC = 0.470, HI = 0.417). The GTR + I + G models were selected as the most effective models for every region of the combined ITS + TEF-1α sequence dataset, and it was used in the Bayesian analysis. The topology obtained from MP and Bayesian analysis was similar to that of ML analysis. The Bayesian analysis yielded a topology that was concordant, with an average split frequency standard deviation of 0.009123. Only the MP tree is shown in Figure 1; bootstrap support values for MP and ML ≥50% and BI ≥0.95 are noted at the nodes, respectively.

The BLAST results showed that the specimen FLF449 (*Mycena glabera*, Holotype) is similar to *M. seminau* (taxon: 1524328), *M. rufobrunnea* (taxon: 2942240), and *M. sinar* (taxon: 1524325), with a sequence similarity of 96.46%–97.83% from NCBI based on ITS with the top 10.

The ITS + TEF-1α phylogenetic tree (Figure 1) confirmed the affinities of *Mycena glabera* in *Mycena* sect. *Calodontes*, and two newly collected specimens formed distinct well-supported lineages (Figure 1).

### 3.2 Taxonomy

**Figure 3:**
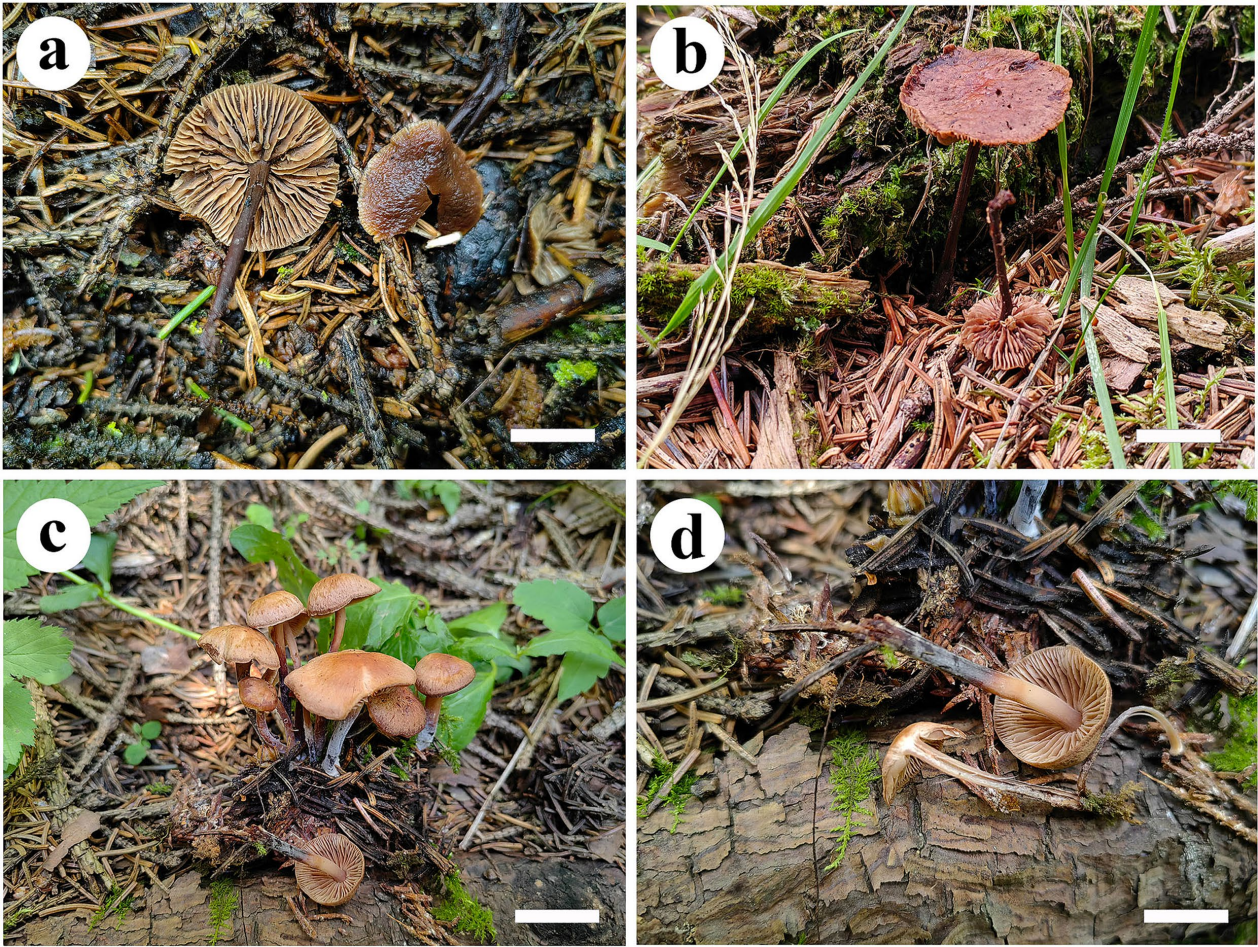
Basidiomata of *Gymnopus* species. *Gymnopus gansuensis* **(a,b)** and *Gymnopus subsepiiconicus* **(c,d)**. Scale bars: 1 cm.

**Figure 4:**
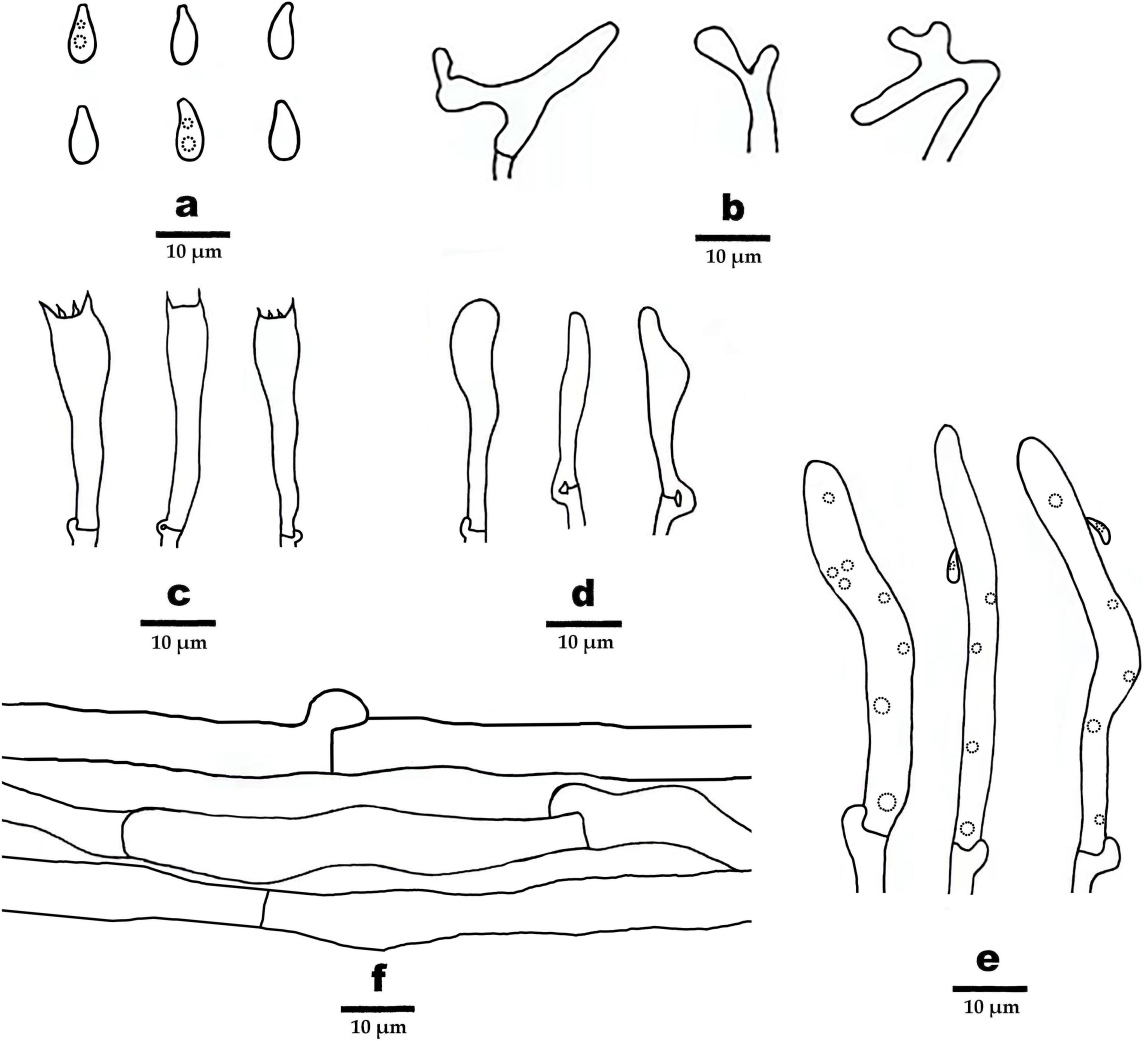
Microscopic structures of *Gymnopus gansuensis* (drawn from the holotype). **(A)** Basidiospores, **(B)** pileipellis, **(C)** basidia, **(D)** basidioles, **(E)** cheilocystidia, and **(F)** hyphae structure.

*Gymnopus gansuensis*, B.Y. Wang, T. F. Ma & L.F. Fan sp. nov. (Figures 3a,b, 4).

MycoBank: 855041.

Etymology: refers to the place of origin in Gansu Province, China.

Diagnosis: Differs from other *Gymnopus* species by pileus honey yellow at the center, margin pinkish buff to buff, stipe pinkish buff to fuscous, and basidiospores elliptic to briolette.

Holotype: CHINA. Gansu Province: Tibetan Autonomous Prefecture of Ganan, Taohe National Nature Reserve, N34°40′70″, E103°53′26″, in coniferous forest, 29 July 2023, MHGAU FLF541.

Basidiomata small-to-medium size, solitary. Pileus usually applanate or slightly convex, 1.0–2.5 cm in diameter, striated, hygrophanus, honey yellow at the center, margin pinkish buff to buff, entire, tomentose near margin. Context thin, fleshy, white to cinnamon buff, odorless. Lamellae subfree to adnate, pinkish buff to honey yellow, crowded. Stipe center, cylindrical, smooth in the upper part, covered with white hairs up to 2/3 (from the base upwards), fistulose, fibrous, 3.2–5.0 cm × 0.2–0.4 cm, pinkish buff near the pileus, fuscous at the bottom.

Hyphal system composed of generative hyphae with clamp connections, IKI–, CB–; tissues turn to black in KOH. Generative hyphae in context light brown to brown, thin-walled, occasionally branched, regularly arranged, 3–8 μm in diameter. Generative hyphae in lamella light brown, thin-walled, occasionally branched, regularly arranged, 5–11 μm in diameter.

Basidiospores elliptic to briolette, smooth, hyaline, IKI–, thin-walled, (6–) 6.5–8 (9) × 3–4 (−4.5) μm, L = 7.5 μm, W = 3.48 μm, Q = 2.16–2.25, (*n* = 60/2). Basidia clavate, smooth, hyaline, thin-walled, 27–35 μm × 6–7 μm, two- or four-spored, sterigmata 1.5–3.5 μm long. Cheilocystidia abundant, clavate, with obtuse at the center, smooth, hyaline, thin-walled, 51–63 μm × 5–6 μm. Pileipellis a cutis, made up of irregularly branched or weakly coralloid hyphae, inflated, smooth, hyaline to light brown, thin-walled, 4–8 μm wide.

Additional specimen (paratype) examined: CHINA. Gansu Province: Tibetan Autonomous Prefecture of Ganan, Taohe National Nature Reserve, N34°40′70″, E103°53′26″, in coniferous forest, 29 July 2023, MHGAU WBY541 (duplicate).

*Gymnopus subsepiiconicus*, B.Y. Wang, T. F. Ma & L.F. Fan sp. nov. (Figures 3c,d).

**Figure 5:**
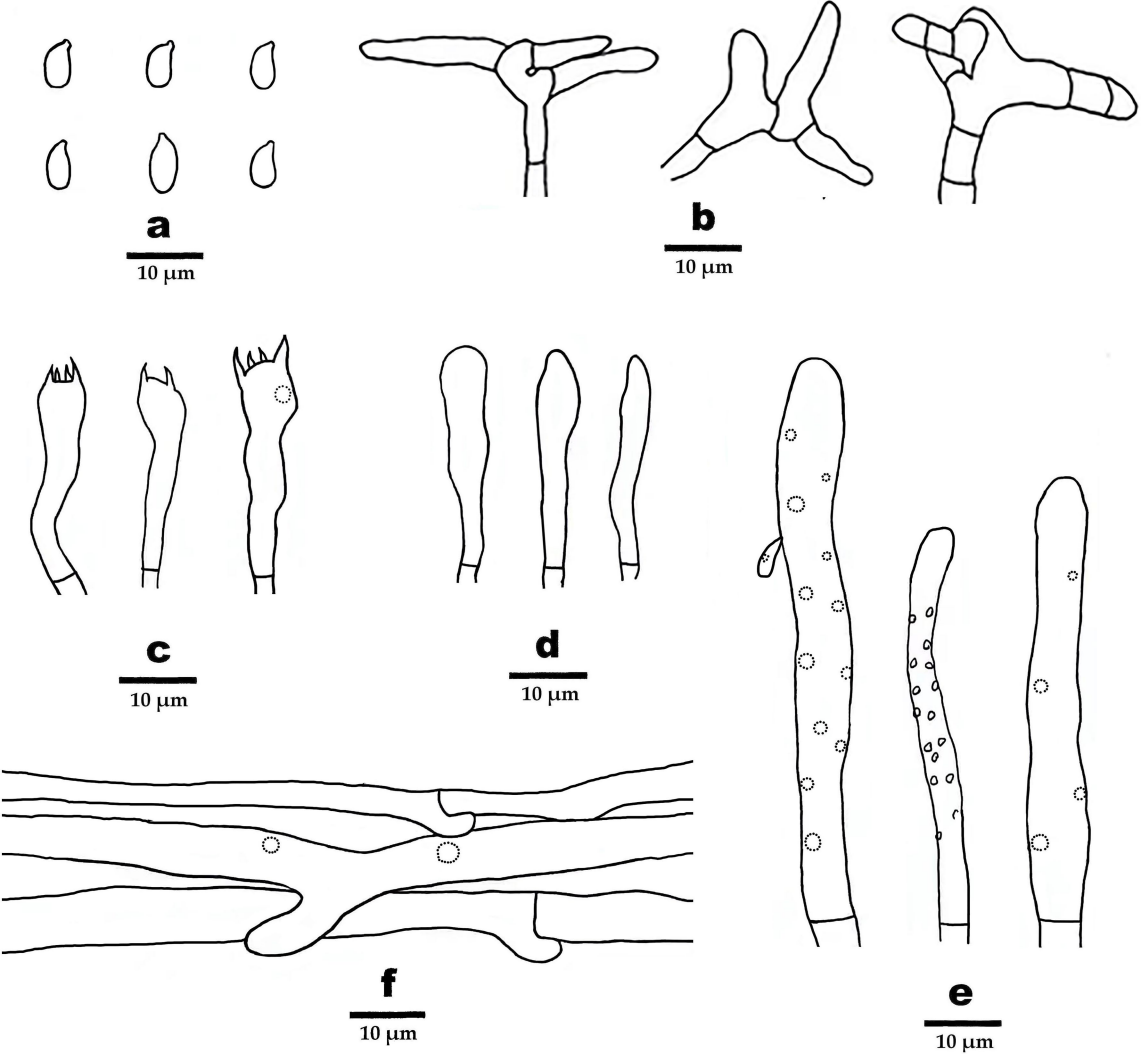
Microscopic structures of *Gymnopus subsepiiconicus* (drawn from the holotype). **(A)** Basidiospores, **(B)** pileipellis, **(C)** basidia, **(D)** basidioles, **(E)** cheilocystidia, and **(F)** hyphae structure.

MycoBank: 855412.

Etymology: refers to the species being similar to *Gymnopus sepiiconicus*.

Diagnosis: Differs from other *Gymnopus* species by pileus clay buff to grayish brown at the center, margin pinkish buff to fawn, stipe dark brown to fuscous, basidiospores elliptic.

Holotype: CHINA. Gansu Province: Lintan County Prefecture of Ganan, Yeliguan National Forest Park, N34°40′70″, E103°53′26″, in coniferous forest land, 27 July 2023, MHGAU FLF393.

Basidiomata in small-to-medium size, solitary to gregarious. Pileus usually applanate or slightly convex, 1.5 cm–2.2 cm in diameter, fluted, hygrophanus, clay buff to grayish brown at the center, margin pinkish buff to fawn, entire. Context thin, fleshy, light grayish brown, odorless. Lamellae subfree to adnate, honey yellow, crowded. Stipe central, cylindrical, 3.2–4.8 cm × 0.2–0.3 cm, grayish brown to dark brown near the pileus, fuscous at the bottom, smooth, fistulose, and fibrous.

Hyphal system composed of generative hyphae with clamp connections, IKI–, CB–; tissues turn to black in KOH. Generative hyphae in context light brown to brown, thin-walled, occasionally branched, regularly arranged, 3 μm –6 μm in diameter. Generative hyphae in lamella grayish brown to dark brown, thin-walled, occasionally branched, regularly arranged, 4 μm–8 μm in diameter.

Basidiospores elliptic, smooth, hyaline, inamyloid, thin-walled, 6–9 × 3–3.5 (−4) μm, L = 7.25 μm, W = 3.25 μm, Q = 2.05–2.4 (*n* = 60/2). Basidia clavate, smooth, hyaline, thin-walled, 19–40 μm × 5–7 μm, two- or four-spored sterigmata 2–5 μm long. Cheilocystidia abundant, clavate, smooth, hyaline, thin-walled, 52–111 μm× 5–6.5 μm. Pileipellis a cutis, made up of irregularly branched or weakly coralloid hyphae, inflated, smooth, hyaline to light brown, thin-walled, 5–15 μm wide.

Additional specimens (paratype) examined: CHINA. Gansu Province: Lintan County Prefecture of Ganan, Yeliguan National Forest Park, N34°56′58.924″, E103°35′47.026″, in coniferous forest, 12 September 2023, MHGAU FLF739, 13 September 2023, MHGAU FLF757.

**Figure 6:**
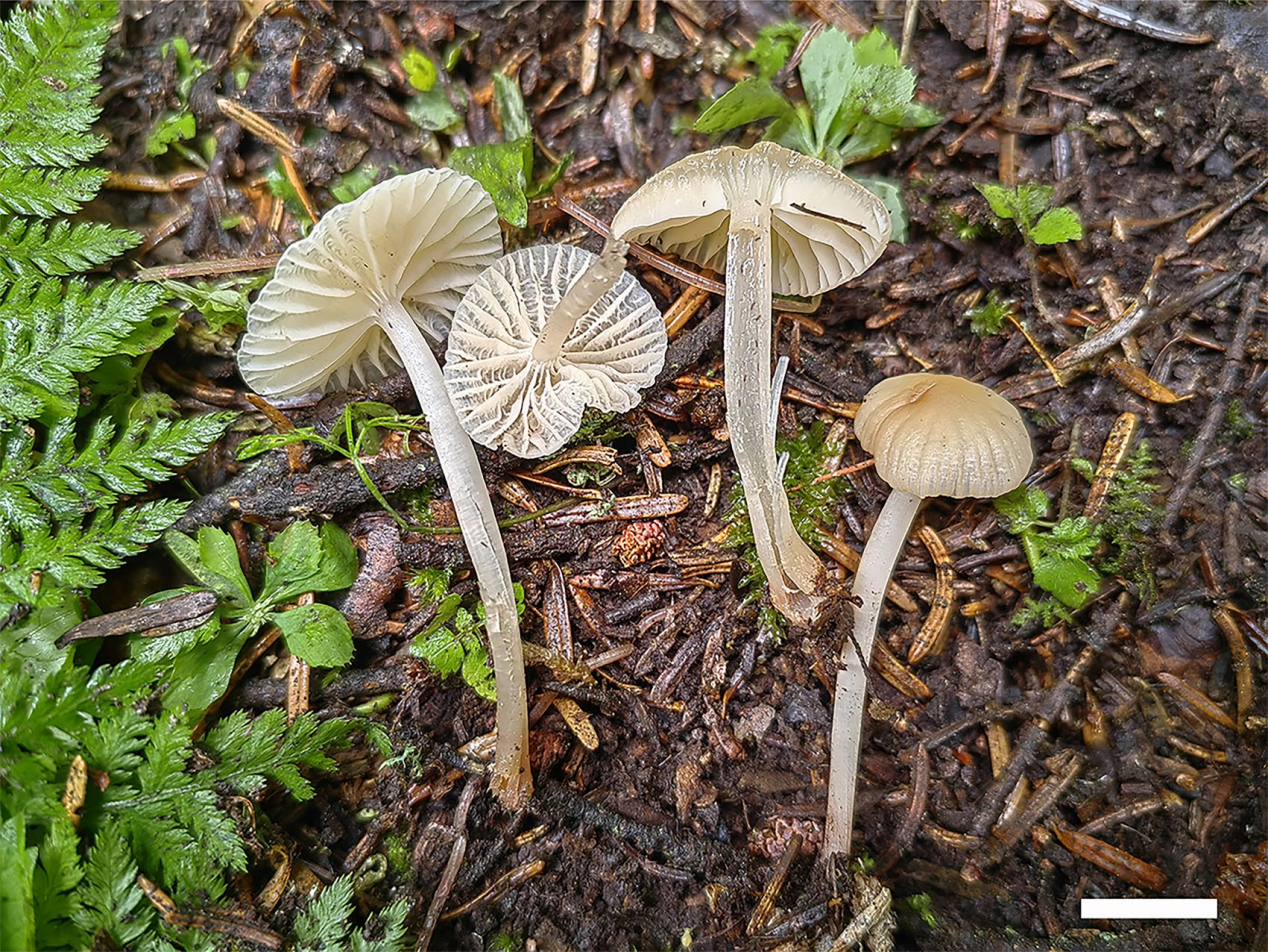
Basidiomata of *Mycena glabera*. Scale bars: 1 cm.

*Mycena glabera*, B.Y. Wang, T. F. Ma & L.F. Fan sp. nov. (Figures 5, 6).

MycoBank: 855042.

Etymology: refers to the smooth and glabrous pileus.

Diagnosis: *Mycena glabera* is characterized by pileus convex or hemispherical, margin decurved, white to cream, slightly wavy, smooth, hygrophanus, surface glabrous, pale pink–yellow to fleshy pink at the center, basidiospores long ellipsoid to cylindrical, thin-walled, cheilocystidia fusiform, thin-walled.

Holotype: CHINA. Gansu Province: Tibetan Autonomous Prefecture of Ganan, Taohe National Nature Reserve, N34°40′70″, E103°53′26″, in coniferous forest, 28 July 2023, MHGAU FLF449.

Basidiomata thin and small, solitary, or scattered. Pileus convex or hemispherical to applanate, margin decurved, white to cream, slightly wavy, smooth, hygrophanus, with vertical stripes or grooves, surface glabrous, old lace to moccasin at the center, 10–20 mm in diameter. Context white, fleshy, very thin (< 1 mm). Lamellae subdecurrent, white to pale pink-yellow, distant. Stipe centrally attached, cylindrical, equal, smooth, fragile, hollow, 30–35 mm × 2–3 mm, white to cream near the pileus, pale wax yellow at the bottom.

Hyphal system composed of generative hyphae with clamp connections, IKI–, CB–; slightly inflated in KOH. Generative hyphae smooth, hyaline, thin-walled, regularly arranged, 3–9 μm in diameter.

Basidiospores long ellipsoid to cylindrical, smooth, hyaline, inamyloid, thin-walled, with obviously oil drop, (5.0–) 6.3–10.2 (−11.7) μm × 3.7–5.5 (−6.7) μm, L = 8.7 μm, W = 4.3 μm, Q = 2.0–2.15, (*n* = 60/2). Basidia clavate, smooth, hyaline, thin-walled, 29.0–45.0 μm × 6.3–12.0 μm, four-spored when mature, sterigmata up to 15.5 μm long. Cheilocystidia abundant, fusiform to subfusiform, with obtuse at the center, smooth, hyaline, thin-walled, 48.0–77.4 μm × 10.0–16.4 μm.

**Figure 7:**
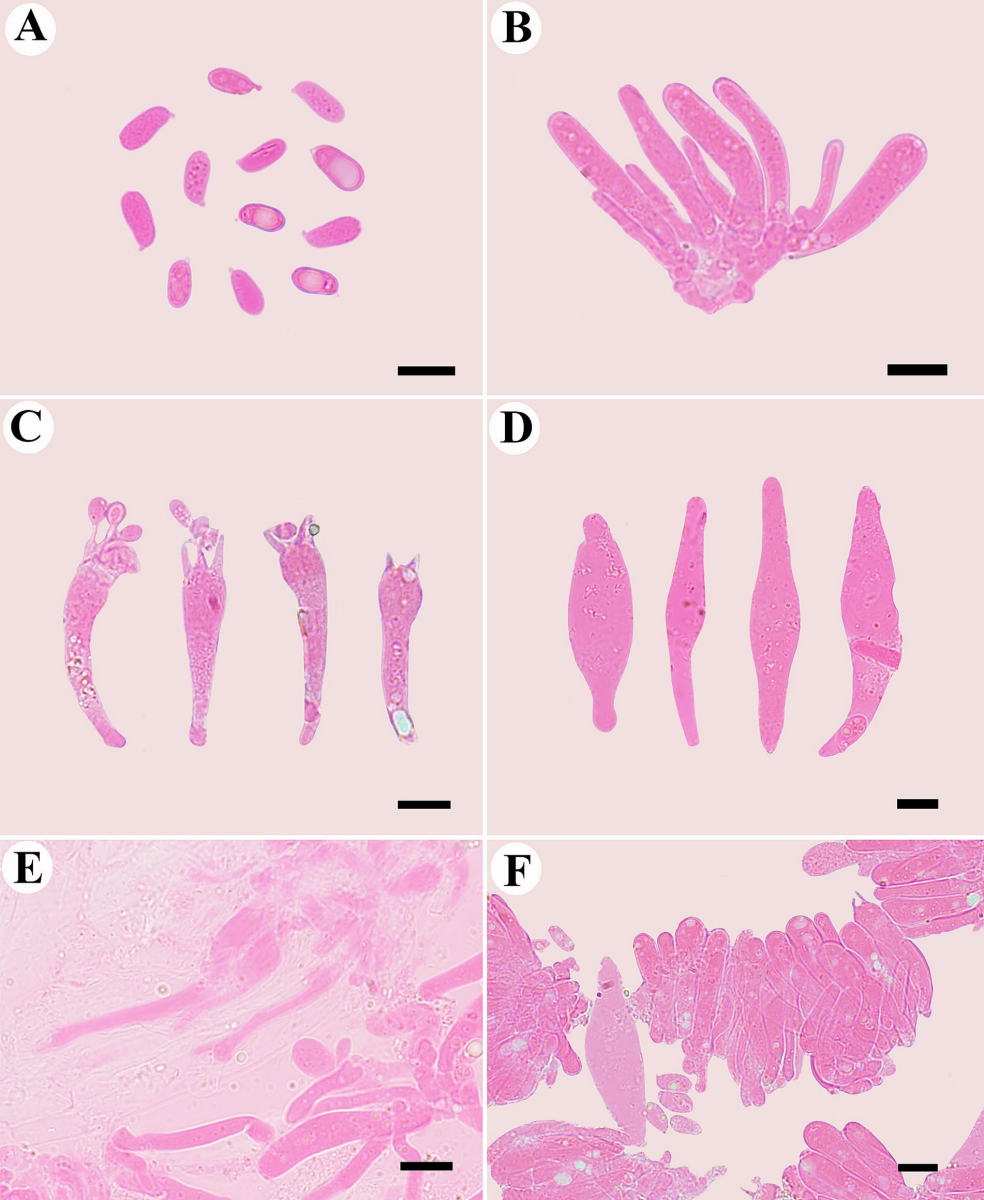
Microscopic structures of *Mycena glabera* (photographed from the holotype). **(A)** Basidiospores, **(B)** probasidia, **(C)** basidia, **(D)** cheilocystidia, **(E)** hyphae with clamp connections, and **(F)** hymenium.

Additional specimen (paratype) examined: CHINA. Gansu Province: Tibetan Autonomous Prefecture of Ganan, Taohe National Nature Reserve, N34°40′70″, E103°53′26″, in coniferous forest, 28 July 2023, MHGAU WBY449 (duplicate) (Figure 7).

## 4 Discussion

Members of *Gymnopus* and *Mycena* are widely dispersed; however, their diversity is not well-recognized in China. In recent years, only three species of *Gymnopus* in China (*G. ramulicola* T.H. Li and S.F. Deng, *G. alliifoetidissimus* T.H. Li and J.P. Li, and *G. pallipes* J.P. Li and Chun Y. Deng) were originally described utilizing molecular evidence. Increased interest in the species variety of *Mycena* has resulted in the publication of numerous new species and significant advancements in scientific understanding. More Chinese scholars focus on the macrofungi in southwest and northeast China; however, the northwest region has received less attention. Phylogenetic analyses revealed that 23 species of *Gymnopus* sect. *Impudicae* (Figure 2) and 33 species of *Mycena* sect. *Calodontes* (Figure 1) were grouped together, respectively, including two new species of *Gymnopus* and one new species of *Mycena* from northwestern China. Our phylogenetic results are consistent with prior studies (Ryoo et al., 2016; Cubeta et al., 1991), and further information on the phylogeny and taxonomy of *Gymnopus* sect. *Impudicae* and *Mycena* sect. *Calodontes* is provided.

Based on our phylogenetic analysis, *G. gansuensis* formed a single branch that was separated from other *Gymnopus* species (Figure 2). Morphologically, *G. similis* is similar to *G. gansuensis* in having brownish orange pileal surface, brownish orange or light brown Lamellae, and similar size of basidiospores measuring 6.5–8.5 μm × 2.7–4 μm (Ryoo et al., 2016). In addition, *G. gansuensis* is characterized by pileus honey yellow at the center, margin pinkish buff to buff, stipe pinkish buff to fuscous, and basidiospores elliptic to briolette. However, *G. similis* differs from *G. gansuensis* by its light to reddish brown to darker (reddish) brown stipe and wider cheilocystidia (20–65 μm × 5–9 μm vs. 51–63 μm × 5–6 μm) (Ryoo et al., 2016).

*Gymnopus subsepiiconicus* is clustered together with *G. aurantiipes* (Corner) A.W. Wilson, Desjardin & E. Horak, *Gymnopus bicolor* A.W. Wilson, Desjardin & E. Horak, and *G. sepiiconicus* (Corner) A.W. Wilson, Desjardin, and E. Horak (Figure 2). Morphologically, *G. aurantiipes* is similar to *G. subsepiiconicus* in having solitary to gregarious basidiomata and convex to applanate pileus (Ronquist and Huelsenbeck, 2003). However, *G. aurantiipes* differs from *G. subsepiiconicus* by its orange pileal surface, yellow pileal margin, yellow to orange–brown to reddish brown stipe, and the smaller basidiospores (4.8–7.2 μm × 2.4–4 μm vs. 6–9 μm × 3–3.5 μm) (Ronquist and Huelsenbeck, 2003). *Gymnopus bicolor* is similar to *G. subsepiiconicus* in having fluted and hygrophanus pileus and central and cylindrical stipe. However, *G. bicolor* differs from *G. subsepiiconicus* by its disk brown pileus, pale orange brown to reddish brown stipe, smaller basidiospores (5.2–8 μm × 2.4–3.6 μm vs. 6–9 μm × 3–3.5 μm), shorter basidia (14.5–22.5 μm × 4–6 μm vs. 19–40 μm × 5–7 μm), and shorter cheilocystidia (17.5–25.5 μm × 6.5–9.5 μm vs. 52–111 μm × 5–6.5 μm) (Ronquist and Huelsenbeck, 2003). *Gymnopus sepiiconicus* is similar to *G. subsepiiconicus* in having convex to applanate pileus and cylindrical stipe. However, *G. sepiiconicus* differs from *G. subsepiiconicus* by its brown to dark brown pileal center, beige yellow to white pileal margin, and shorter basidiospores (4.8–6.4 μm × 2.4–4.4 μm vs. 6–9 μm × 3–3.5 μm) (Ronquist and Huelsenbeck, 2003; Wilson et al., 2004).

*Mycena glabera*, sistered with *M. rufobrunnea* Z.W. Liu, Y.P. Ge & Q., is grouped together with *M. seminau* A.L.C. Chew & Desjardin and *M. sinar* A.L.C. Chew & Desjardin (Figure 1) and indistinguishable in the shape or size of basidiospores. However, *M. rufobrunnea* can differ from *M. glabera* by having dark brown pileus at the center, grayish-magenta to dull violet stipe in the upper part, and utriform cheilocystidia (Cortés-Pérez et al., 2023; Liu et al., 2022). *Mycena seminau* is similar to *M. glabera* in having elongate to cylindrical, smooth, hyaline, and thin-walled basidiospores. However, *M. seminau* differs from *M. glabera* by its brown to dark brown pileus at the center, smaller basidia (20.8–25.6 μm × 4.8–8.0 μm vs. 29.0–45.0 μm × 6.3–12.0 μm) and basidiospores (7.6–8.8 μm × 3.6–4.4 μm vs. 6.3–10.2 μm × 3.7–5.5 μm) (Chew et al., 2014). *Mycena glabera* is similar to *M. sinar* in having elongate to cylindrical basidiospores and similar in size and subdecurrent lamellae. However, *M. sinar* can be differentiated from *M. glabera* by having brownish orange to yellowish brown pileus at the center, narrower basidia (4.8–8.8 vs. 6.3–12.0) and abundant, fusiform to subfusiform cheilocystidia (Chew et al., 2014).

The species diversity of *Gymnopus* and *Mycena* from China has been increased after the comprehensive research. This study provided a basis for further research on *Gymnopus* sect. *Impudicae* and *Mycena* sect. *Calodontes*. However, the systematic research of the genera was restricted because only a small number of *Gymnopus* sect. *Impudicae* and *Mycena* Sect. *Calodontes* species with multiple genes accessible could be used for the analysis. For the time being, the most effective DNA barcoding for the identification of *Gymnopus* and *Mycena* species is ITS, while more samples with multigene sequences, including mt-SSU, RPB1, and RPB2, are needed to further investigate the species diversity and phylogenetic relationships of mushroom-forming species.

## Data Availability

The datasets presented in this study can be found in online repositories. The names of the repository/repositories and accession number(s) can be found in the article/supplementary material.

## Footnotes

^1^https://www.indexfungorum.org/

^2^https://www.mycobank.org/
